# Writing the Signs: An Explainable Machine Learning Approach for Alzheimer's Disease Classification from Handwriting

**DOI:** 10.1049/htl2.70006

**Published:** 2025-02-13

**Authors:** Ngoc Truc Ngan Ho, Paulina Gonzalez, Gideon K. Gogovi

**Affiliations:** ^1^ Department of Computer Science Lehigh University Bethlehem Pennsylvania USA; ^2^ Department of Population Health Lehigh University Bethlehem Pennsylvania USA; ^3^ Department of Biostatistics and Health Data Science Lehigh University Bethlehem Pennsylvania USA

**Keywords:** Alzheimer's disease, ensemble learning, explainability, handwriting, SHAP

## Abstract

Alzheimer's disease is a global health challenge, emphasizing the need for early detection to enable timely intervention and improve outcomes. This study analyzes handwriting data from individuals with and without Alzheimer's to identify predictive features across copying, graphic and memory‐based tasks. Machine learning models, including Random Forest, Bootstrap Aggregating (Bagging), Extreme Gradient Boosting (XGBoost), Light Gradient Boosting Machine (LightGBM), Adaptive Boosting (AdaBoost) and Gradient Boosting, were applied to classify patients, with SHapley Additive exPlanations (SHAP) enhancing model interpretability. Time‐related features were crucial in copying and graphic tasks, reflecting cognitive processing speed, while pressure‐related features were significant in memory tasks, indicating recall confidence. Simpler graphic tasks showed strong discriminatory power, aiding early detection. Performance metrics demonstrated model effectiveness: For memory tasks, Random Forest achieved the highest accuracy (0.840±0.038), while Bagged SVC was the lowest (0.617±0.046). Copying tasks recorded a peak accuracy of 0.804±0.075 with Gradient Boost and a low of 0.566±0.032 for Bagged SVC. Graphic tasks reached 0.799±0.041 with Gradient Boost and 0.643 ± 0.071 with AdaBoost. For all tasks combined, Random Forest excelled (0.854±0.033), while Gradient Boost performed worst (0.598±0.151). These results highlight handwriting analysis's potential in Alzheimer's detection.

## Graphical abstract

Alzheimer's disease poses a major global health challenge, necessitating early detection for better treatment outcomes. This study analyzed handwriting data from various tasks using explainable ensemble models, revealing that time‐related features were significant in copying and graphic tasks, while pressure‐related features were important in memory tasks. SHapley Additive exPlanations (SHAP) enhanced model interpretability, highlighting the potential of handwriting features to aid in early detection of cognitive decline.

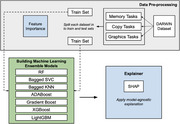

## 1 Introduction

Alzheimer's disease (AD) represents one of the most formidable global health challenges, with its impact extending to millions of individuals worldwide. As a progressive neurodegenerative disorder, AD is characterized by significant memory loss, cognitive impairment and behavioural changes, which progressively worsen over time. At a cellular level, the accumulation of beta‐amyloid plaques and hyperphosphorylated tau protein tangles within the brain disrupt synaptic communication between neurons, leading to widespread neurodegeneration, inflammation and cerebral atrophy [1]. These neuropathological changes are strongly linked to the cognitive and functional declines observed in AD patients. Over time, individuals with AD become increasingly dependent on care‐giving, ultimately requiring full‐time assistance in the advanced stages of the disease.

AD accounts for 60%–70% of all dementia cases globally, placing a profound burden on both healthcare systems and the families of affected individuals [2]. Between 1990 and 2019, the global incidence of AD surged by approximately 148%, underscoring the need for strategies to promote early detection and intervention [3]. Timely diagnosis plays a crucial role in initiating treatments that can slow disease progression, alleviate symptoms and improve patients' quality of life. Early detection also provides patients and their families with valuable time to make informed decisions about future care and participate in therapeutic interventions that may enhance well‐being [4, 5].

Achieving early diagnosis of AD remains a significant challenge. Clinicians often encounter obstacles such as under recognition of early symptoms, societal stigma surrounding dementia and difficulties in effectively communicating a diagnosis to patients and their families [4]. Additionally, existing diagnostic techniques, which rely heavily on neuroimaging and neuropsychological testing, are expensive, time‐intensive and frequently inaccessible in resource‐limited settings [6]. These limitations highlight the critical need for more accessible, cost‐effective and reliable diagnostic tools to facilitate early identification of AD.

In recent years, machine learning (ML) has emerged as a powerful tool for advancing early diagnosis in various healthcare applications, including AD. ML models have been applied to diverse multimodal data sources, such as neuroimaging and clinical assessments, significantly enhancing diagnostic accuracy and disease progression prediction [7, 8]. For instance, models utilizing data from functional MRI and neuropsychological assessments have demonstrated high accuracy in distinguishing AD from its precursor, mild cognitive impairment (MCI), which is critical for timely intervention [7, 8]. Beyond AD, ML and artificial intelligence methods have shown promise in detecting pediatric foot deformities using plantar pressure measurements [9], early diagnosis of Parkinson's disease through deep learning and ML techniques [10] and unsupervised COVID‐19 infection detection using deep generative learning‐based 1‐SVM detectors on blood test data [11].

In addition to neuroimaging, handwriting analysis has emerged as a promising non‐invasive diagnostic tool for detecting AD. Handwriting tasks, which engage complex cognitive and motor processes, are often disrupted in individuals with AD. Research indicates that handwriting features, such as slower movements, tremors, and irregularities, may serve as early indicators of cognitive decline [12, 13]. Extensive research has demonstrated the potential of handwriting datasets in improving AD diagnostic performance using various methods. For instance, reference [12] implemented a weighted ensemble combining logistic regression and support vector machines (SVMs), significantly improving early‐stage AD prediction from analyzing handwriting. Ensemble methods thus hold promise in overcoming the limitations of single‐model approaches by offering enhanced predictive power and robustness in AD diagnosis. Another study focused on explainability in convolutional neural network (CNN)‐based AD detection from online handwriting data, emphasizing the importance of understanding the decision‐making process of CNN models by providing explainable outputs for AD diagnosis [14]. An ML approach analyzed handwriting features extracted through the sigma‐lognormal model, demonstrating that changes in these features can serve as indicators of AD. This highlights the potential for using quantitative handwriting features to classify individuals with AD [15]. Another study developed an ML‐powered handwriting analysis framework for the early detection of AD, leveraging ML techniques to detect subtle differences in handwriting patterns that are often indicative of cognitive decline, reinforcing the potential of handwriting as an early biomarker for AD [16].

An in‐depth analysis of handwriting characteristics assessed AD and MCI by examining a range of kinematic features. The study provided evidence that handwriting analysis could distinguish AD patients, offering valuable insights into how cognitive decline affects motor functions [17]. Other research investigated kinematic features of handwriting to differentiate between AD and normal aging, focusing on specific writing deficits observed in AD patients compared to elderly individuals without cognitive impairments [18]. A similar study analyzed differences in handwriting kinematic characteristics between AD patients and healthy elderly individuals, aiming to determine whether handwriting analysis could be used as an auxiliary screening tool or as part of an AD diagnostic system. The findings provided a foundation for the development of handwriting‐based diagnostic tools, further establishing the relevance of handwriting analysis in the context of AD early detection [19].

Even though ML models have proven their utility in various medical applications, their opacity presents significant challenges in clinical practice. As a result, many healthcare settings prefer using more straightforward, interpretable statistical models, such as linear regression, despite their limitations in predictive accuracy [20]. Although there has been substantial work focused on interpreting complex models and opening the “black box” of ML decision‐making [21, 22], relatively few recent studies have concentrated on improving the interpretability and explainability of ML models in the context of AD. To be accepted and trusted by clinicians, these models need to be both transparent and retraceable, allowing for a clear understanding of their role in clinical decision‐making. Clinicians require models that not only provide accurate predictions but also offer interpretable insights that can inform decision‐making [22, 23, 24]. Therefore, it is essential for ML models to clearly explain the medical decisions or diagnostic tasks they perform. In this regard, a recent study presented an explainable AI (XAI) approach [25] that utilized structural analysis of AD. This research employed a modified deep BrainNet model to classify individuals with AD, MCI and NC. Through ablation analysis, the study identified key brain regions and their connectivity related to AD by evaluating how these regions and connections impact the model's predictions and visualizing the brain areas that most strongly drive its outputs. The study by reference [26] applied XAI to a multimodal data fusion model, providing interpretable predictions of AD progression, while reference [23] integrated explainable ensemble learning to identify key factors contributing to AD diagnosis.

Three explainable deep learning architectures proposed by reference [27] focused on analyzing language capabilities for detecting AD. Each model utilized different feature sets: part‐of‐speech attributes, language embeddings or a hybrid approach combining both. Two types of explanations were generated: intra‐class explanations, which provide insights into the significance of individual features within the same class, and inter‐class explanations, which contrast feature importance across different classes. Similarly, reference [28] presented another explainable deep learning framework for diagnosing AD. In another study, reference [29] leveraged ML to examine significant factors contributing to AD progression, employing an XGBoost (Extreme Gradient Boosting) model to classify disease stages. SHapley Additive exPlanations (SHAP) were used alongside the trained model to provide both local and global interpretability. Additionally, a SHAP model was used together with neural networks [30] to classify AD using handwriting data and employed a SHAP model combined with an RF classifier to categorize cognitive impairment and dementia using cognitive scores as inputs [31].

Handwriting analysis has shown potential in diagnosing AD, but prior studies often relied on ML models with limited interpretability, restricting their clinical applicability. This study advances the field by leveraging explainable ensemble methods–such as (RF), Bagged SVC, Bagged k‐Nearest Neighbors (KNN), Adaptive Boosting (ADABoost), XGBoost, and Light Gradient Boosting Machine (LightGBM)–augmented with SHAP to classify AD using handwriting data from memory, copying and graphic tasks. Unlike traditional black‐box models, our approach emphasizes interpretability while maintaining high diagnostic accuracy. Task‐specific insights reveal the importance of time‐related metrics in copying and graphic tasks and pressure‐related features in memory tasks, providing actionable information for clinical decision‐making.

Additionally, this study compares ensemble models, highlighting their robustness and efficiency. While acknowledging the trade‐offs between interpretability and the potential of deep learning methods to capture complex patterns, our findings underscore the practical value of explainable ensemble models in healthcare. By focusing on time‐related, pressure‐related and movement‐related handwriting features, this study sheds light on how cognitive impairment affects handwriting behaviour, offering actionable insights and advancing the application of interpretable ML in AD research.

## 2 Method

### 2.1 Data Description

The Diagnosis AlzheimeR WIth haNdwriting (DARWIN) dataset, introduced by reference [32], offers a valuable resource for AD detection through handwriting analysis. While no single dataset can entirely resolve the challenge of limited data availability, DARWIN represents an important step in addressing this gap by providing a structured collection of handwriting data from individuals with AD and healthy controls. The dataset supports further research aimed at improving the accuracy of diagnostic models, particularly in early detection, through the analysis of cognitive and motor impairments captured in handwriting tasks. The dataset comprises data from 174 participants—89 diagnosed with AD and 85 healthy controls. The participants were selected based on standard clinical assessments, including the Mini‐Mental State Examination (MMSE), Frontal Assessment Battery (FAB) and Montreal Cognitive Assessment (MoCA). The dataset consists of 450 unique features for each data point, making it both compact and highly complex due to its high dimensionality. The AD group consisted of 46 women and 44 men, with an average age of 71.5 (±9.5) years and an average education level of 10.8 (±5.1) years. The healthy control group included 51 women and 39 men, with an average age of 68.9 (±12) years and an average education level of 12.9 (±4.4) years.

Data collection for the DARWIN dataset was conducted using a WACOM Bamboo Folio graphic tablet and pen, according to the structured protocol outlined by [32]. The protocol comprised 25 handwriting tasks, each tailored to assess various cognitive and motor skills critical for diagnosing Alzheimer's Disease. We classified the tasks into three categories, based on their distinct cognitive and motor demands. The first category, Graphic tasks, focused on evaluating basic motor skills through activities like drawing geometric shapes, connecting points, and retracing lines. The second category, Copy and Reverse Copy tasks, assessed participants' ability to replicate complex gestures, including writing letters, words, and numbers. Last, the third category, Memory and Dictation tasks, tested participants' memory recall as well as their ability to write specific words, phrases, or numbers based on dictation or memory prompts.

This carefully curated dataset provides valuable insights into handwriting behaviours associated with AD, supporting the development of advanced diagnostic models in neurodegenerative research. Eighteen distinct features, as detailed in Table 1, were extracted from the handwriting data for each task, focusing on three key areas: motor control, pressure dynamics and temporal characteristics. These features provided comprehensive insights into various aspects of handwriting behaviour, including the total time taken to complete each task, writing speed, pen pressure and indicators of tremor. Together, these features enabled a detailed analysis of both cognitive and motor impairments associated with AD, supporting the development of more accurate diagnostic models.

**TABLE 1:** Summary of Features Extracted from the Handwriting Data.

Variable	Description
**Time‐related**	
total_time	Total time spent to perform the entire task.
air_time	Time spent to perform in‐air movements.
paper_time	Time spent to perform on‐paper movements.
**Movement‐related**	
mean_speed_on_paper	Average speed of on‐paper movements.
mean_speed_in_air	Average speed of in‐air movements.
mean_acc_on_paper	Average acceleration of on‐paper movements.
mean_acc_in_air	Average acceleration of in‐air movements.
mean_jerk_on_paper	Average jerk of on‐paper movements.
mean_jerk_in_air	Average jerk of in‐air movements.
gmrt_on_paper	Generalization of Mean Relative Tremor (GMRT) for on‐paper movements.
gmrt_in_air	GMRT computed for in‐air movements.
mean_gmrt	Average of gmrt_on_paper and gmrt_in_air.
num_of_pendown	Total number of times the pen touched the paper.
max_x_extension	Maximum horizontal distance covered during writing.
max_y_extension	Maximum vertical distance covered during writing.
disp_index	The extent of trace spread across the sheet of paper.
**Pressure‐related**	
pressure_mean	Average of the pressure levels exerted by the pen tip.
pressure_var	Variance of the pressure levels exerted by the pen tip.

The features extracted from the handwriting data were selected for their potential to capture relevant motor and cognitive changes associated with AD. These features, categorized into time‐related, movement‐related and pressure‐related groups (as shown in Table 1), were chosen to reflect impairments in cognitive processing, motor control and handwriting dynamics, all of which are commonly affected in AD.

Time‐related features such as total time spent on the task, time spent on in‐air movements and time spent on paper movements were selected for their ability to capture the speed of cognitive and motor processes. Cognitive decline in AD often leads to slower processing speeds, resulting in longer task completion times or increased time spent lifting the pen. These metrics are critical for assessing cognitive processing speed and motor coordination.

Movement‐related features such as average speed, average acceleration and generalization of mean relative tremor (GMRT) for on‐paper movements were included to assess motor control. These features help evaluate the speed, acceleration and tremor of handwriting movements, all of which are typically impacted in AD. Changes in these movement characteristics, such as slower speeds or irregular acceleration, may indicate cognitive or motor impairments. Specifically, the inclusion of GMRT is important for identifying tremor or jitter that may result from cognitive and motor dysfunction in AD.

The pressure‐related features, the average pressure exerted by the pen tip and the variance in pressure levels, were chosen to assess motor control during handwriting. In AD patients, inconsistent or fluctuating pressure may signal difficulty in maintaining fine motor control, as cognitive decline affects both the planning and execution of movements. These pressure metrics offer valuable insights into motor confidence and control during handwriting tasks. Together, these features capture key handwriting characteristics influenced by cognitive and motor changes in AD. They are well‐suited to identify early signs of cognitive decline and offer significant potential for Alzheimer's disease detection through handwriting analysis.

### 2.2 Machine Learning Methods

This study leveraged a range of ML algorithms to enhance predictive accuracy and model robustness. The chosen methods—RF, Bootstrap Aggregating (Bagging), Gradient Boosting, XGBoost, LightGBM, and AdaBoost—are prominent ensemble techniques known for their efficacy in handling classification tasks. Each method contributed unique strengths to the analysis.

RF is an ensemble learning method that constructs multiple decision trees as base learners [33]. Each tree is trained on a bootstrap sample of the dataset, with a random subset of features selected at each node to determine the optimal split [34]. The final prediction is based on majority voting across all trees, reducing overfitting and fostering diversity among the trees. By employing impurity measures, such as Gini impurity and entropy, the algorithm ensures that splits maximize impurity reduction at each node [35].

Bootstrap Aggregating, or Bagging, is another ensemble technique aimed at reducing variance and mitigating overfitting by training multiple base classifiers on different bootstrap samples of the data [36]. The final prediction is determined by majority voting across the classifiers. In this study, KNN and SVMs were used as base classifiers. Bagging enhances robustness by reducing model variance, particularly when combined with high‐variance classifiers [37]. Gradient Boosting builds an ensemble of weak learners in a sequential manner, correcting errors of preceding models by fitting to the negative gradient of the loss function [38]. This approach allows the model to optimize complex loss functions effectively. Regularization techniques such as limiting tree depth, feature sub‐sampling and early stopping were implemented to prevent overfitting and improve generalization [39].

XGBoost extends traditional Gradient Boosting by incorporating both first‐ and second‐order gradients (Hessians), enhancing loss function approximation [40]. Regularization techniques, including L1 and L2 penalties, and subsampling during training further reduced overfitting. The learning rate was carefully tuned to control the contribution of each tree, enhancing convergence and generalization. LightGBM, a highly efficient Gradient Boosting framework, was utilized to accelerate training and improve prediction accuracy [41]. Key features include Gradient‐based One‐Side Sampling (GOSS) and Exclusive Feature Bundling (EFB), which prioritize informative samples and reduce dimensionality, respectively. Although the dataset was not high‐dimensional, these techniques improved training speed and memory efficiency. LightGBM's leaf‐wise tree growth strategy, which optimizes loss reduction more effectively than level‐wise methods, further boosted accuracy [42].

Finally, AdaBoost sequentially trains weak learners, focusing on misclassified instances from previous iterations [43]. By iteratively adjusting instance weights, AdaBoost emphasizes difficult cases, allowing the ensemble to iteratively improve its performance. This process builds a strong classifier from a series of weak learners, making it particularly effective for classification tasks. These ML techniques collectively enhanced the study's predictive capabilities by leveraging their strengths in handling classification challenges. Each method was carefully implemented and optimized to ensure robust and accurate modelling outcomes.

### 2.3 Machine Learning Explanations With SHAP

To validate the rules generated by our ML models and assess the explanatory power of individual features in AD classification, we applied SHAP. SHAP is a model‐agnostic method for interpreting the predictions of ML models by attributing the contribution of each feature to individual predictions. This capability provides insights into the decision‐making process of complex models, ensuring transparency and interpretability [44].

Rooted in cooperative game theory, SHAP is based on Shapley values, which were originally developed to fairly distribute payouts in multi‐player games. In the context of ML, SHAP assigns importance scores to features based on their contribution to a model's prediction for a specific data point. This enables a detailed breakdown of how each feature influences the output for an individual sample, offering instance‐level interpretability.

Mathematically, SHAP decomposes a model's prediction for a given sample, $x_{i}$, into contributions from individual features as follows:

$${\hat{y}}_{i}=y_{\text{base}}+\sum_{j=1}^{n}f\left(x_{ij}\right),$$

where $y_{\text{base}}$ is the average prediction across all samples (serving as a baseline or reference point) and $f(x_{ij})$ is the SHAP value associated with the jth feature of the ith sample. A positive SHAP value indicates that the feature increases the prediction relative to the baseline, while a negative SHAP value indicates a decrease.

By quantifying the contribution of features such as time‐related and pressure‐related variables, SHAP allows us to understand how specific features influence predictions for AD classification. Additionally, SHAP is uniquely suited for explaining predictions at the individual level, which is particularly valuable for personalized healthcare applications. For instance, understanding why a specific participant is classified as having AD can aid clinicians in corroborating model predictions with clinical evidence. The transparency offered by SHAP helps build trust in the model, supporting actionable decision‐making in clinical settings.

SHAP adheres to three essential properties that ensure fairness and consistency in feature attributions. The first, local accuracy (Additivity), ensures that the sum of all SHAP values for a prediction equals the difference between the predicted value and the baseline prediction, accurately reflecting the model's behaviour. The second, consistency (Symmetry), guarantees that if a feature's contribution to the model increases, its SHAP value will also increase, ensuring consistency in feature importance rankings. Finally, the Null Effect property ensures that features that do not influence the prediction are assigned a SHAP value of zero, preventing spurious attributions.

In this study, SHAP was used to analyze the contributions of handwriting‐related features to the ML models' predictions. The application of SHAP in this context not only validated the discriminatory power of these features but also provided a nuanced understanding of how they influence AD classification. This interpretability enhances the clinical utility of the ML models by ensuring that predictions are explainable and grounded in meaningful feature contributions.

### 2.4 Ensemble Model Development

In this study, we developed and evaluated several ensemble models to assess their performance across the multiple datasets categorizations. The ensemble methods employed include RF, Bagging, XGBoost, LightGBM, AdaBoost, and Gradient Boosting, all chosen for their ability to handle diverse data structures while employing explainable artificial intelligence methods to provide a level of interpretability in their predictions. The models were applied to the four distinct dataset categories, each representing different task categories. The first dataset consisted of all 25 activities across various tasks, providing a comprehensive foundation for model evaluation. The second dataset focused specifically on copying‐related tasks, allowing for targeted analysis of performance in this area. The third dataset emphasized memory recall and citation tasks, testing the models on their ability to retain information and accurately reference it. Finally, the fourth dataset was dedicated to graphic‐related tasks, assessing the models' performance in visual and graphical capabilities.

**FIGURE 1:**
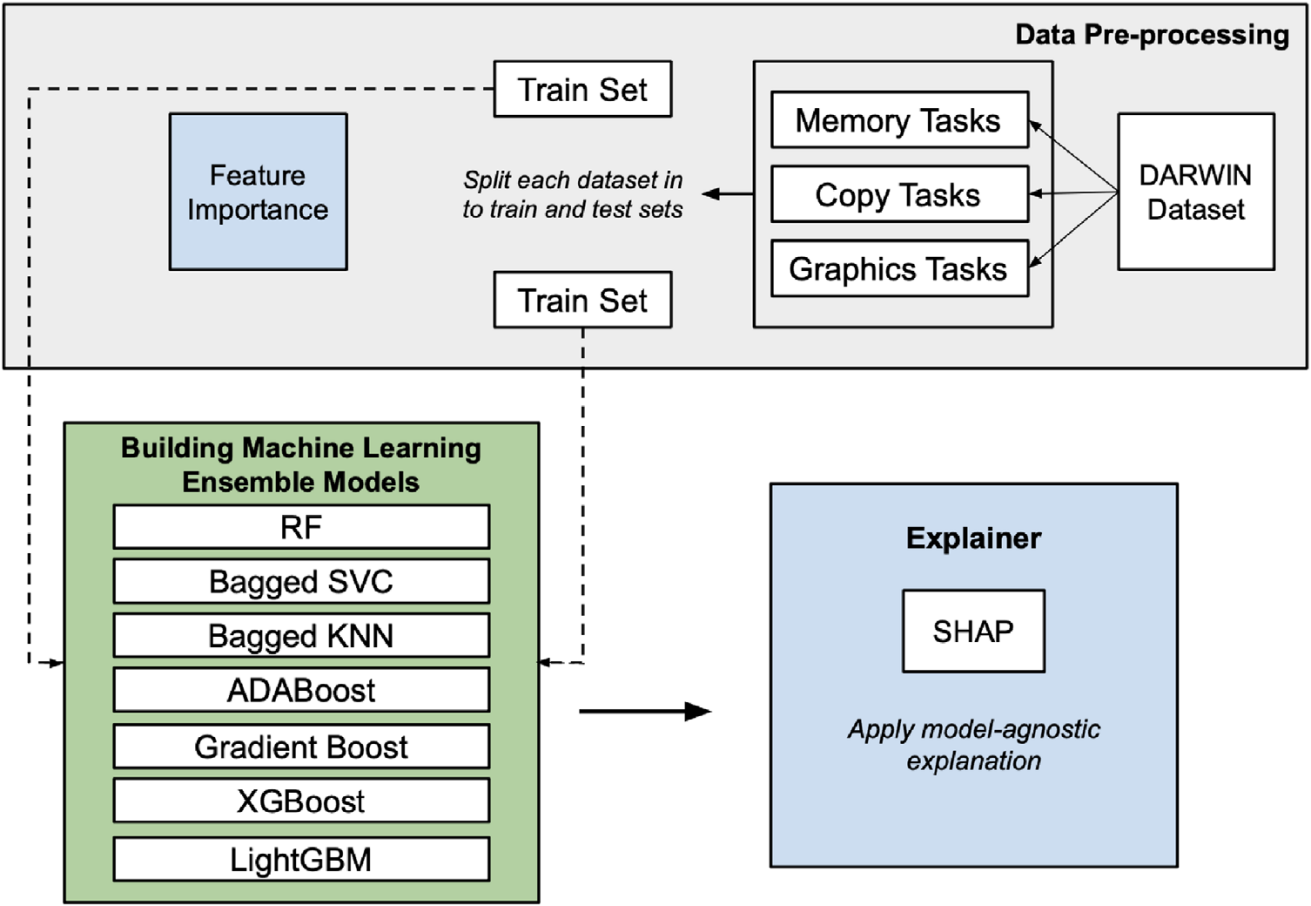
Overview of the proposed methodology, starting with data acquisition from the UCI Machine Learning Repository, followed by preprocessing, feature importance evaluation, ensemble classifier construction and applying model‐agnostic explanations to interpret AD‐related factors.

To ensure a robust evaluation of model performance, we employed a standard train‐test split strategy. Each dataset was divided into a training set, comprising 75% of the data, and a testing set, comprising the remaining 25%. This approach allowed the models to be trained on the majority of the data while ensuring unbiased performance evaluation on the unseen test samples. The performance of each ensemble model was optimized through careful hyperparameter tuning. For RF, we adjusted the number of trees and tree depth while keeping other parameters at their default settings. In Bagging, we optimized the number of base estimators and sampling parameters. With XGBoost, we fine‐tuned hyperparameters such as learning rate, maximum tree depth and the number of estimators to enhance its gradient‐boosting performance. Adjustments to LightGBM involved modifying the learning rate and the number of leaves and boosting iterations to efficiently handle large‐scale datasets. For AdaBoost, we focused on optimizing the number of estimators and the learning rate, while Gradient Boosting saw fine‐tuning of the learning rate, number of estimators and tree depth to maximize performance. The protocol outlining the overall methodology is shown in Figure 1.

### 2.5 Hyperparameter Tuning

We conducted 20 independent training and evaluation cycles for each proposed hyperparameter configuration to account for model performance variability due to factors like random initialization and data shuffling. During this process, we calculated average performance metrics—such as accuracy, sensitivity, specificity, and F1 score—for both training and testing datasets. This step was critical in identifying overfitting or underfitting issues. Once the optimal hyperparameter set was identified, a final evaluation was performed using fivefold cross‐validation [45]. Each subset served as the test set while the remaining data were used for training, providing a thorough performance assessment, with results averaged across all folds. These outcomes offered robust performance estimates, reported as mean values with corresponding standard deviations.

This methodology was consistently applied across models to ensure a fair and comprehensive comparison of their abilities to handle the datasets. It not only optimized individual model performance but also provided insights into each model's strengths and weaknesses. Hyperparameter tuning was facilitated by Optuna, an open‐source framework for efficient optimization, leveraging Bayesian optimization via the Tree‐structured Parzen Estimator (TPE) algorithm [46]. The search space covered a broad range of hyperparameter configurations, enabling us to thoroughly evaluate each model and enhance its predictive performance. A grid search was employed within defined hyperparameter ranges to systematically evaluate different configurations, optimizing performance across the datasets. This exhaustive search through the hyperparameter space allowed us to assess the impact of each parameter, ensuring the models performed optimally under the varying conditions presented by different task categories. The final parameter combinations for each model are presented in Table 2.

**TABLE 2:** Parameter ranges explored during optimization.

Model	All tasks	Copy tasks	Memory tasks	Graphic tasks
Random Forest	criterion: gini, entropy, log_loss max_depth: [2, 15], step = 1 n_estimator: [100, 550], step=10 bootstrap: True, False min_samples_split: [2, 20], step=1 min_sample_leaf: [1, 20], step=1 max_features: [0.1, 1.0], step=0.045	criterion: gini, entropy, log_loss max_depth: [2, 5] n_estimator: [3, 175] bootstrap: True, False min_samples_split: [20, 50] min_sample_leaf: [20, 50], step=1 max_features: [0.1, 1.0], step=0.045	criterion: gini, entropy, log_loss max_depth: [2, 5] n_estimator: [3, 175] bootstrap: True, False min_samples_split: [20, 50] min_sample_leaf: [20, 50], step=1 max_features: [0.1, 1.0], step=0.045	criterion: gini, entropy, log_loss max_depth: [2, 5] n_estimator: [3, 175] bootstrap: True, False min_samples_split: [20, 50] min_sample_leaf: [20, 50], step=1 max_features: [0.1, 1.0], step=0.045
Bagged SVC	n_estimators: [70, 350] max_samples: [0.8, 1.0] max_features: [0.5, 1.0] oob_score: True, False	n_estimators: [150, 450] max_samples: [0.5, 1.0] max_features: [0.4, 1.0] oob_score: True, False	n_estimators: [150, 450] max_samples: [0.5, 1.0] max_features: [0.4, 1.0] oob_score: True, False	n_estimators: [150, 450] max_samples: [0.5, 1.0] max_features: [0.4, 1.0] oob_score: True, False
Bagged KNN	n_estimators: [50, 200] max_samples: [0.5, 1.0] max_features: [0.5, 1.0] bootstrap: True, False	n_estimators: [100, 200] max_samples: [0.5, 1.0] max_features: [0.5, 1.0] bootstrap: True, False	n_estimators: [100, 200] max_samples: [0.5, 1.0] max_features: [0.5, 1.0] bootstrap: True, False	n_estimators: [100, 200] max_samples: [0.5, 1.0] max_features: [0.5, 1.0] bootstrap: True, False
XGBoost	learning_rate: [1e‐5, 1e‐3] max_depth: [2, 4] min_child_weight: [1, 10] subsample: [0.6, 0.8] colsample_bytree: [0.2, 0.5] gamma: [5, 10], lambda: [5, 10] alpha: [5, 10], n_estimators: [250, 400] objective: binary:logistic eval_metric: logloss	learning_rate: [1e‐5, 1e‐4] max_depth: [2, 4] min_child_weight: [1, 10] subsample: [0.6, 0.8] colsample_bytree: [0.2, 0.5] gamma: [5, 10], lambda: [5, 10] alpha: [5, 10], n_estimators: [250, 400] objective: binary:logistic eval_metric: logloss	learning_rate: [1e‐5, 1e‐4] max_depth: [2, 4] min_child_weight: [1, 10] subsample: [0.6, 0.8] colsample_bytree: [0.2, 0.5] gamma: [5, 10], lambda: [5, 10] alpha: [5, 10], n_estimators: [250, 400] objective: binary:logistic eval_metric: logloss	learning_rate: [1e‐2, 1.0] max_depth: [2, 6] min_child_weight: [1, 10] subsample: [0.6, 0.8] colsample_bytree: [0.2, 0.5] gamma: [1, 8], lambda: [1, 8] alpha: [1, 8], n_estimators: [200, 500] objective: binary:logistic eval_metric: logloss
AdaBoost	n_estimators: [20, 150] learning_rate: [1e‐8, 1e‐2] algorithm: [”SAMME”, ”SAMME.R”] base_estimator params: max_depth: [1, 5] min_samples_split: [2, 20] min_samples_leaf: [1, 10]	n_estimators: [5, 30] learning_rate: [1e‐8, 1e‐2] algorithm: [”SAMME”] base_estimator params: max_depth: [1, 5] min_samples_split: [2, 20] min_samples_leaf: [1, 10]	n_estimators: [5, 30] learning_rate: [1e‐8, 1e‐2] algorithm: [”SAMME”] base_estimator params: max_depth: [1, 5] min_samples_split: [2, 20] min_samples_leaf: [1, 10]	n_estimators: [5, 30] learning_rate: [1e‐8, 1e‐2] algorithm: [”SAMME”] base_estimator params: max_depth: [1, 5] min_samples_split: [2, 20] min_samples_leaf: [1, 10]
Gradient Boost	n_estimators: [250, 400] learning_rate: [1e‐6, 1e‐4] max_depth: [2, 7] min_samples_split: [4, 18] min_samples_leaf: [4, 18] subsample: [0.6, 0.8] max_features: [0.2, 0.5] min_weight_fraction_leaf: [0.0, 0.5] n_iter_no_change: [8, 15] min_impurity_decrease: [0.0, 0.1]	n_estimators: [100, 400] learning_rate: [1e‐5, 1e‐3] max_depth: [2, 15] min_samples_split: [2, 15] min_samples_leaf: [2, 15] subsample: [0.6, 0.8] max_features: [0.2, 0.5] min_weight_fraction_leaf: [0.0, 0.4] n_iter_no_change: [5, 10] min_impurity_decrease: [0.0, 0.05]	n_estimators: [100, 400] learning_rate: [1e‐5, 1e‐3] max_depth: [2, 15] min_samples_split: [2, 15] min_samples_leaf: [2, 15] subsample: [0.6, 0.8] max_features: [0.2, 0.5] min_weight_fraction_leaf: [0.0, 0.4] n_iter_no_change: [5, 10] min_impurity_decrease: [0.0, 0.05]	n_estimators: [100, 400] learning_rate: [1e‐5, 1e‐3] max_depth: [2, 15] min_samples_split: [2, 15] min_samples_leaf: [2, 15] subsample: [0.6, 0.8] max_features: [0.2, 0.5] min_weight_fraction_leaf: [0.0, 0.4] n_iter_no_change: [5, 10] min_impurity_decrease: [0.0, 0.05]
LightGBM	feature_pre_filter: False objective: binary metric: binary_logloss boosting_type: gbdt lambda_l1: [3.0, 28.0] lambda_l2: [3.0, 28.0] num_leaves: [8, 30] feature_fraction: [0.4, 1.0] bagging_fraction: [0.4, 1.0] bagging_freq: [1, 7] min_child_samples: [3, 24] min_data_in_bin: [3, 20] min_split_gain: [0.0, 0.5] max_depth: [2, 23] learning_rate: [1e‐4, 0.1]	feature_pre_filter: False objective: binary metric: binary_logloss boosting_type: gbdt lambda_l1: [2.5, 16.0] lambda_l2: [2.5, 16.0] num_leaves: [2, 20] feature_fraction: [0.4, 1.0] bagging_fraction: [0.3, 1.0] bagging_freq: [1, 7] min_child_samples: [3, 30] min_data_in_bin: [12, 20] min_split_gain: [0.0, 0.5] max_depth: [2, 15] learning_rate: [1e‐2, 8.0]	feature_pre_filter: False objective: binary metric: binary_logloss boosting_type: gbdt lambda_l1: [1e‐8, 10.0] lambda_l2: [1e‐8, 10.0] num_leaves: [5, 70] feature_fraction: [0.5, 1.0] bagging_fraction: [0.5, 1.0] bagging_freq: [1, 7] min_child_samples: [3, 15] min_data_in_bin: [10, 20] min_split_gain: [0.0, 0.6] max_depth: [2, 45] learning_rate: [1e‐9, 0.7]	feature_pre_filter: False objective: binary metric: binary_logloss boosting_type: gbdt lambda_l1: [3.0, 13.0] lambda_l2: [3.0, 13.0] num_leaves: [3, 20] feature_fraction: [0.5, 1.0] bagging_fraction: [0.5, 0.8] bagging_freq: [1, 7] min_child_samples: [3, 20] min_data_in_bin: [12, 20] min_split_gain: [0.0, 0.6] max_depth: [2, 15] learning_rate: [1e‐2, 5.0]

### 2.6 Evaluation of the Classification Ensemble Model

To evaluate the performance of the ensemble ML models, we utilized three primary metrics: accuracy, sensitivity and specificity. These metrics assess the model's effectiveness in distinguishing between patients with AD and healthy individuals.

*Accuracy* measures the proportion of correctly predicted cases, both positive and negative. Mathematically, it is defined as:

$$\text{Accuracy}=\frac{\text{TP}+\text{TN}}{\text{TP}+\text{TN}+\text{FP}+\text{FN}}$$

where TP represents True Positives (correctly identified AD cases), TN denotes True Negatives (correctly identified healthy cases), FP indicates False Positives (incorrectly identified AD cases), and FN refers to False Negatives (incorrectly identified healthy cases). This metric provides a general measure of the model's performance across all classes.

*Sensitivity*, also known as the True Positive Rate or Recall, evaluates the model's ability to accurately identify individuals with Alzheimer's Disease. It is computed as:

$$\text{Sensitivity}=\frac{\text{TP}}{\text{TP}+\text{FN}}$$

This metric highlights the model's capacity to minimize false negatives, ensuring that most individuals with AD are correctly recognized. High sensitivity signifies effective detection of true AD cases.

*Specificity*, or the True Negative Rate, assesses the model's ability to correctly identify individuals who do not have AD. It is expressed as:

$$\text{Specificity}=\frac{\text{TN}}{\text{TN}+\text{FP}}$$

This metric reflects the model's capability to avoid false positives, ensuring accurate classification of healthy individuals. High specificity indicates the model's proficiency in distinguishing non‐AD cases.

## 3 Results

The results obtained from the various data categorizations are presented in detail. For each category, the outcomes from all classifiers are included, allowing for a comprehensive comparison. Detailed metrics for each classifier are provided to facilitate an evaluation of their performance across the corresponding data categorizations are described as follows.

### 3.1 Ensemble ML Method Performance Comparison in Memory Tasks

The evaluation of ensemble models in memory tasks revealed their superior performance in classifying AD patients. Among the models assessed, RF achieved the highest accuracy of (83.96±3.78)%, demonstrating strong sensitivity at (90.15±8.85)% but relatively lower specificity at (78.67±9.89)%. XGBoost and AdaBoost displayed identical performance, both achieving an accuracy of (83.90±3.91)%, with a sensitivity at (90.31±8.91)% and specificity at (78.39±10.20)%. LightGBM followed closely with an accuracy of (83.33±2.83)%, showcasing a more balanced performance with sensitivity of (89.31±9.16)% and the same specificity as XGBoost and AdaBoost.

The consistently high sensitivity across all models suggests that memory tasks are particularly effective in identifying individuals with cognitive impairment or AD, making these tasks invaluable for early detection. Although the models slightly favour sensitivity, they maintain reasonable specificity, averaging around 78%–80% for the top performers. This balance highlights their capability to detect AD while minimizing false positives, an essential factor for reliable classification. Additionally, the strong and consistent performance across various models indicates that the features derived from memory tasks are robustly informative, thereby enhancing the reliability of this diagnostic approach. The performance comparison for memory‐related tasks is presented in Table 3 below.

**TABLE 3:** Model performance: Memory and copy tasks.

	Memory tasks			Copy tasks		
	Accuracy	Sensitivity	Specificity	Accuracy	Sensitivity	Specificity
Random Forest	0.840 ± 0.038	0.901 ± 0.089	0.787 ± 0.099	0.785 ± 0.054	0.774 ± 0.078	0.801 ± 0.113
Bagged SVC	0.617 ± 0.046	0.420 ± 0.130	0.834 ± 0.151	0.566 ± 0.032	0.997 ± 0.005	0.114 ± 0.048
Bagged KNN	0.698 ± 0.094	0.524 ± 0.159	0.885 ± 0.072	0.750 ± 0.104	0.579 ± 0.182	0.929 ± 0.041
XGBoost	0.781 ± 0.078	0.903 ± 0.089	0.673 ± 0.194	0.763 ± 0.087	0.874 ± 0.123	0.676 ± 0.255
AdaBoost	0.839 ± 0.039	0.903 ± 0.089	0.784 ± 0.102	0.701 ± 0.072	0.712 ± 0.094	0.697 ± 0.132
LightGBM	0.833 ± 0.028	0.893 ± 0.092	0.784 ± 0.102	0.764 ± 0.055	0.749 ± 0.086	0.773 ± 0.117
Gradient Boost	0.822 ± 0.025	0.885 ± 0.113	0.773 ± 0.131	0.804 ± 0.075	0.903 ± 0.088	0.720 ± 0.208

### 3.2 Ensemble Method Performance Comparison in Copy Tasks

In the realm of copy tasks, RF emerged as the leading model, achieving an accuracy of (78.49±5.38)%. This model demonstrated balanced performance with a sensitivity of (77.41±7.75)% and specificity of (80.13±11.26)%. The consistency across these performance metrics indicates RF's robustness in managing the features associated with copy tasks. XGBoost exhibited strong sensitivity at (87.42±12.30)%, but it showed lower specificity at (67.64±25.53)%, resulting in an overall accuracy of (76.35±8.72)%. This suggests that XGBoost may be more aggressive in identifying positive cases, potentially increasing the likelihood of false positives. The higher standard deviations in performance metrics imply that copy tasks may demonstrate less reliability across different patient groups or datasets.

Although copy tasks do not achieve the same level of consistent effectiveness as memory tasks, they still provide valuable diagnostic insights. The balance between sensitivity and specificity, particularly evident in RF, indicates that these tasks can play a significant role in both identifying potential cases and ruling out true negatives. The variability in model performance highlights the critical importance of careful model selection when evaluating copy tasks. These tasks are especially useful for assessing specific cognitive functions, making them essential for differential diagnosis and monitoring disease progression. The performance comparison for copy tasks is presented in Table 3.

### 3.3 Ensemble Method Performance Comparison in Graphic Tasks

Graphic tasks performance comparison, as presented in Table 4, proved to be the most challenging category across all models. Bagged SVC emerged as the top performer with an accuracy of (77.56±4.08)%. It exhibited a strong bias toward specificity at (89.55±7.31)% over sensitivity at (66.48±5.68)%, suggesting its effectiveness in correctly identifying negative cases but a potential weakness in capturing all positive cases. XGBoost followed closely with an accuracy of (77.53±7.34)%, demonstrating a more balanced performance between sensitivity at (76.27±5.28)% and specificity at (79.64±13.50)%. RF achieved an accuracy of (75.29±4.80), with a sensitivity of (69.05±9.04) and specificity of (82.47±10.64). Its lower performance compared to other task categories suggests that RF may find the features from graphic tasks more challenging to interpret effectively. The high specificity, particularly in Bagged SVC, indicates that graphic tasks can be especially useful in ruling out AD and reducing false positives. While displaying lower overall performance, graphic tasks may capture unique aspects of cognitive functions that are not reflected in other tasks. The performance comparison for copy tasks is presented in Table 4 below.

**TABLE 4:** Model Performance: Graphic and All Tasks.

	Graphic Tasks			All Tasks		
Model	Accuracy	Sensitivity	Specificity	Accuracy	Sensitivity	Specificity
Random Forest	0.753 ± 0.048	0.691 ± 0.090	0.825 ± 0.106	0.854 ± 0.033	0.886 ± 0.083	0.832 ± 0.091
Bagged SVC	0.776 ± 0.041	0.665 ± 0.057	0.895 ± 0.073	0.607 ± 0.082	0.976 ± 0.025	0.229 ± 0.163
Bagged KNN	0.743 ± 0.028	0.618 ± 0.072	0.879 ± 0.078	0.762 ± 0.107	0.587 ± 0.198	0.947 ± 0.041
XGBoost	0.775 ± 0.073	0.763 ± 0.053	0.796 ± 0.135	0.850 ± 0.045	0.891 ± 0.073	0.819 ± 0.104
AdaBoost	0.643 ± 0.071	0.564 ± 0.099	0.742 ± 0.208	0.839 ± 0.039	0.903 ± 0.089	0.784 ± 0.102
LightGBM	0.701 ± 0.038	0.672 ± 0.140	0.729 ± 0.127	0.828 ± 0.018	0.883 ± 0.098	0.784 ± 0.102
Gradient Boost	0.799 ± 0.041	0.841 ± 0.086	0.771 ± 0.130	0.598 ± 0.151	0.877 ± 0.161	0.355 ± 0.398

### 3.4 Ensemble Method Performance Comparison for All Tasks

RF demonstrated the highest accuracy across all tasks, achieving (85.40±3.35)%. This model also exhibited a strong balance between sensitivity at (88.56±8.32)% and specificity at (83.19±9.06)%. The consistent superior performance of RF across multiple task categories underscores its versatility and robustness in effectively managing diverse features. In comparison, XGBoost and AdaBoost performed well, particularly in terms of sensitivity, but did not quite match the accuracy levels of RF. XGBoost achieved an accuracy of (85.01±4.46)%, with sensitivity at (89.11±7.30)% and specificity at (81.86±10.37)%. AdaBoost recorded an accuracy of (83.90±3.91)%, sensitivity of (90.31±8.91)% and specificity of (78.39±10.20)%. While these models excelled in sensitivity, RF's superior accuracy highlights its overall effectiveness in handling the diverse nature of the tasks. The results, as shown in Table 4, suggest that while sensitivity is crucial for identifying potential cases, the balance of sensitivity and specificity demonstrated by RF makes it particularly valuable for accurate classification. Overall, the ensemble models showed varying performance across different tasks, emphasizing the importance of model selection based on the specific characteristics of the data and the objectives of the analysis.

### 3.5 Comparison of State‐of‐the‐Art Methods

Table 5 compares our top‐performing models with state‐of‐the‐art approaches that utilized the same dataset, evaluating key metrics such as accuracy, sensitivity, specificity, and F1 score. The results highlight the strengths of our models in enabling the early detection of AD. Specifically, our RF model achieves an accuracy of 85.4% with a standard deviation of 3.3%, which is competitive with methods like CNN and LGBM. This performance underscores the model's reliability in accurate AD detection. In terms of sensitivity, the Bagged SVC model achieves the highest score of 97.6%, significantly surpassing other methods, including traditional ML models such as SVM and RF, demonstrating its ability to effectively identify AD cases. Additionally, the Bagged KNN model attains the highest specificity of 94.7%, outperforming all other approaches and minimizing false positives, which is crucial for maintaining diagnostic accuracy. These results emphasize the effectiveness of our models in detecting AD while maintaining a strong balance between sensitivity and specificity, showcasing their potential for clinical applications.

**TABLE 5:** Comparison with state‐of‐the‐art methods across all 25 handwriting tasks. Bold text denotes the highest score.

Method	Accuracy	Sensitivity	Specificity
VGG19 [47]	75.0	75.0	—
Mobile NetV2 [47]	59.6	59.6	—
InceptionV3 [47]	76.9	76.9	—
SVM [32]	79.0	77.5	80.6
RF [32]	88.3± 5.0	90.3	86.2
LGBM [47]	88.5	95.8	—
CNN [47]	**90.4**	90.4	—
Proposed ensemble models	85.4 ± 3.3(RF)	**97.6 ± 2.5** (Bagged SVC)	**94.7 ± 4.1** (Bagged KNN)

### 3.6 Model Explanations With SHapley Additive exPlanations

The SHAP waterfall plots offer a comprehensive visual representation of the contributions of each feature in predicting AD across various ensemble models and task types. Positive SHAP values indicate a feature's contribution toward an AD classification, while negative SHAP values suggest a tendency toward a healthy classification.

When examining the models' performance across all tasks—copy tasks, graphic tasks and memory tasks—distinct patterns emerge regarding the influential features. These patterns become even more pronounced in task‐specific analyses, providing a nuanced understanding of how different features interact and influence model predictions. This approach enhances our insight into the underlying mechanisms driving the classifications, ultimately informing more targeted strategies for diagnosis and intervention.

**FIGURE 2:**
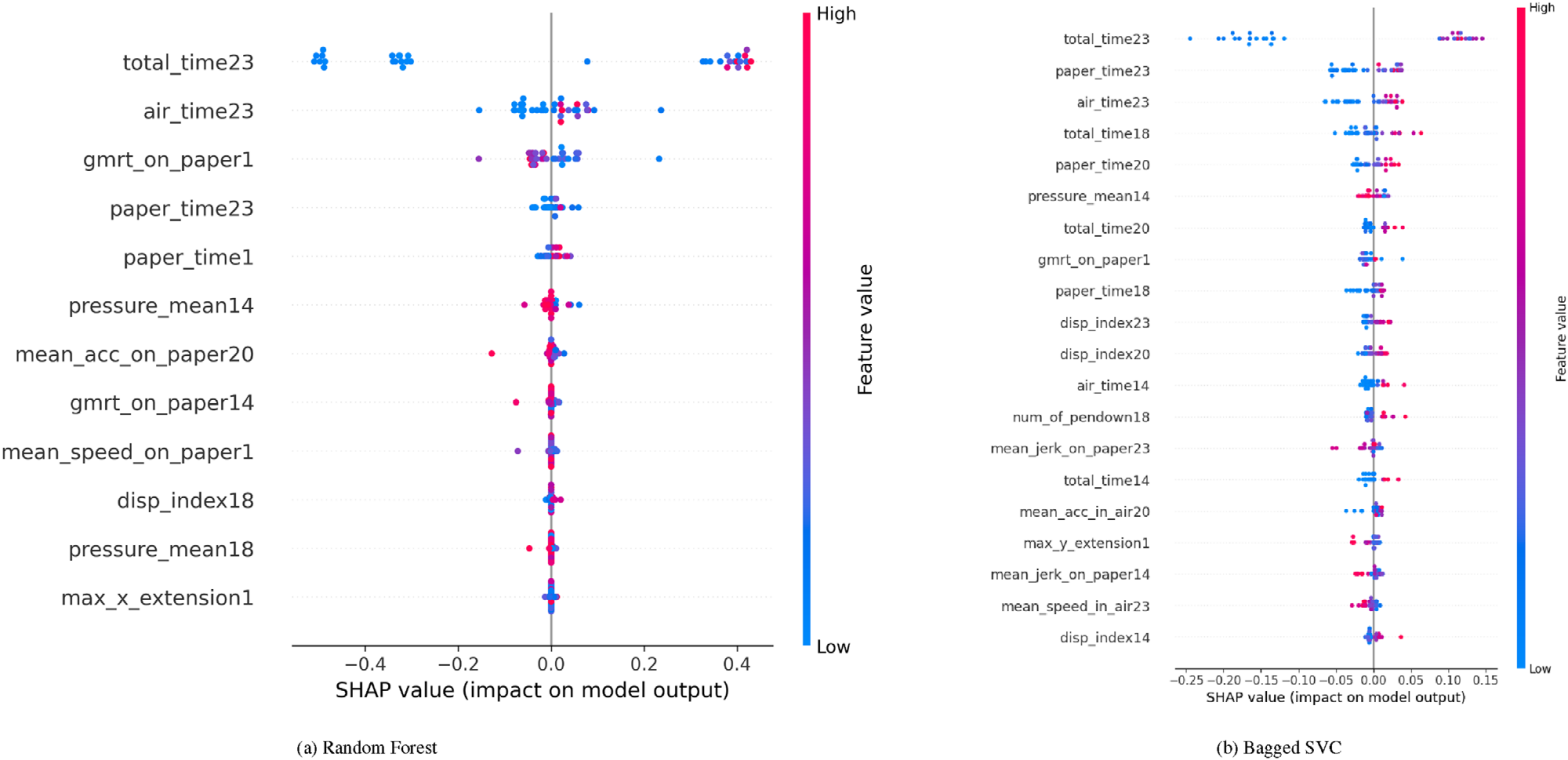
Explanation provided by the SHAP model for all tasks. (a) Random Forest; (b) Bagged SVC.

In the all‐tasks category, the RF model highlighted task 23, which involves writing a telephone number under dictation, as the most influential across the models (see Figure 2a). The total time spent on this task consistently yields the highest SHAP values, emphasizing the significance of time‐related features—such as air time and paper time—in predicting AD. The evidence is clear: Longer completion times for tasks correlate strongly with a higher likelihood of classifying a participant as having AD. This trend persists across most models, indicating a robust relationship between task duration and cognitive decline associated with the disease.

In contrast, the Bagged SVC model, as shown in Figure 2b, identifies pressure variance in task 3, which requires joining two points with a vertical line, as its most influential feature. This finding suggests that while time‐related features are critical for many of the models, pressure‐based indicators of fine motor control also play a significant role in distinguishing between AD and healthy classes. The Bagged KNN model aligns with this observation, emphasizing pressure variance in task 9 as the most important feature. Here, pressure‐related metrics gain prominence, although time features continue to significantly contribute to the predictions of these models. Models such as XGBoost, Gradient Boost and LightGBM reflect the performance of RF by demonstrating a strong dependence on total time in task 23. These models predominantly rely on positive SHAP values associated with time‐related features, reinforcing the notion that extended task durations are indicative of Alzheimer's progression. The consistent appearance of these time‐related features across multiple ensemble models further underscores their critical importance in predicting AD.

**FIGURE 3:**
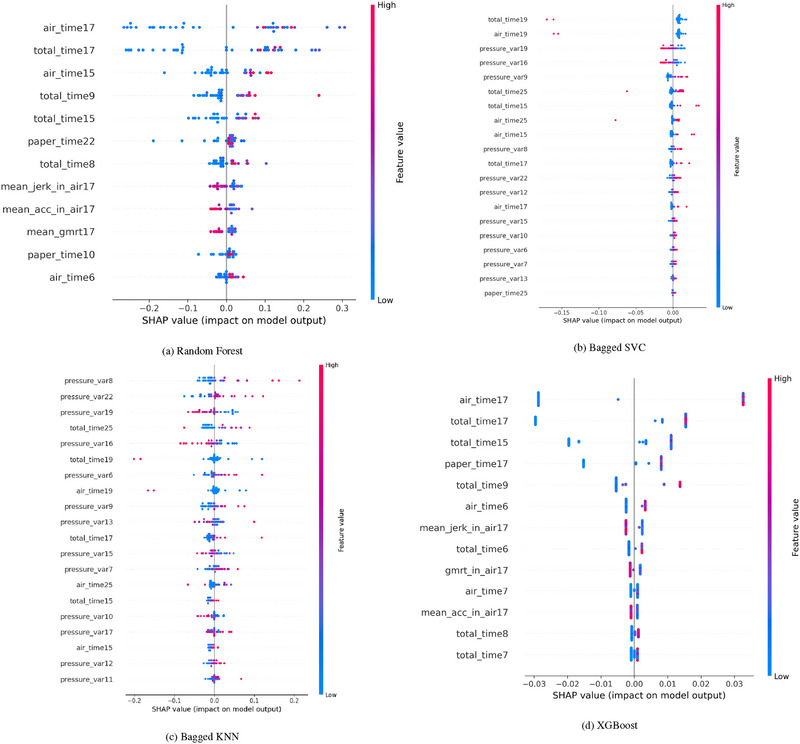
Explanation provided by the SHAP model for copy tasks. (a) Random Forest; (b) Bagged SVC; (c) Bagged KNN; (d) XGBoost.

The RF model, as shown in Figure 3a, highlights air time as the most influential feature, with positive SHAP values contributing to predictions toward AD classification in the copy tasks category. In task 17, where participants copy six words, air time emerges as a dominant feature, reflecting the amount of time participants spend planning before initiating the writing process. Longer air times are strongly associated with cognitive decline, indicating hesitation or difficulty in task planning—common symptoms of AD. The model's predictions are predominantly influenced by positive SHAP values linked to these temporal features. However, some negative SHAP values associated with features such as mean jerk in the air and mean acceleration reveal nuanced aspects of the data, suggesting that variations in how AD manifests in these tasks can differ across individuals.

The Bagged SVC model identifies total time in task 19, where participants copy a postal order, as the most critical feature, with negative SHAP values influencing predictions (see Figure 3b). This may indicate that certain outliers or edge cases in the data—such as shorter task completion times—are more related to participant‐specific factors rather than the progression of AD. Pressure‐related features remain significant, with pressure variance once again playing an important role in guiding the model's decision‐making process.

Similarly, the Bagged KNN model in Figure 3c emphasizes pressure variance, particularly in task 8, which involves writing a sequence of lowercase “i”s in cursive. This finding suggests that fine motor control, measured through the variability in applied pressure during writing, is crucial for distinguishing between AD and healthy classifications in copy tasks. Variability in pressure during task execution likely reflects underlying cognitive and motor coordination difficulties, both of which are key indicators of AD.

For XGBoost, air time in task 17 remains a high‐impact feature, further underscoring the significance of planning time in AD diagnosis. The Gradient Boosting model focuses heavily on total time for task 17, while LightGBM adopts a different approach, identifying mean jerk in the air during task 17 as the most influential feature, with negative SHAP values standing out. This suggests that for certain models, the smoothness and control of movements, rather than task duration, are more critical in predicting cognitive decline, particularly in tasks that demand fine motor skills.

**FIGURE 4:**
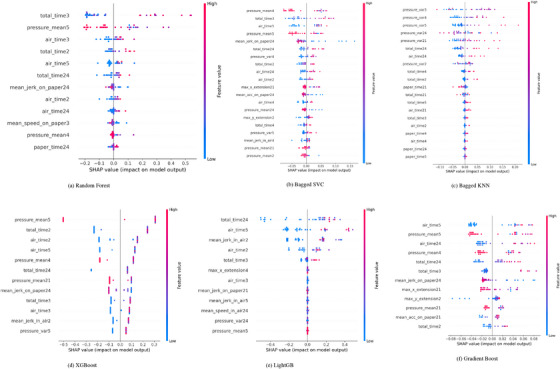
Explanation provided by the SHAP model for graphics tasks. (a) Random Forest; (b) Bagged SVC; (c) Bagged KNN; (d) XGBoost; (e) LightGB; (f) Gradient Boost.

In graphic tasks, which primarily assess motor control and spatial awareness, the RF model places the greatest emphasis on total time for task 3 (as illustrated in Figure 4a), which involves joining two points with vertical lines. As with other task categories, time‐related features remain crucial, with positive SHAP values driving predictions toward an AD classification. The Bagged SVC model, shown in Figure 4b, focuses on the mean pressure exerted in task 4, where participants retrace a circle. Here, both positive and negative SHAP values influence the model, suggesting that pressure features can impact predictions in either direction, depending on the participant's specific performance. This contrasts with time features, which tend to consistently have a positive impact on AD classification. Pressure‐related features exhibit greater variability, reflecting the complex role of motor control in these tasks. Figure 4c presents the SHAP values of the Bagged KNN model, which further underscores the significance of pressure‐related features, particularly pressure variance in task 3, highlighting its critical role in the performance of graphic tasks. In the XGBoost, pressure variance during task 5, which involves drawing alternating squares and triangles, emerges as the most influential feature (see Figure 4d). The distribution of both positive and negative SHAP values for pressure‐related features suggests that the fine motor control required to complete these tasks varies significantly between participants, adding complexity to the model's predictions. LightGBM, in Figure 4e, similarly highlights total time in task 24, the Clock Drawing Test, as the most important feature, reinforcing the consistent relevance of task duration across all models when predicting AD patients based on graphic tasks. This consistency in task duration as a predictor, juxtaposed with the variability in pressure features, reflects the multi‐dimensional nature of motor control and spatial awareness in AD classification. The Gradient Boosting model placed greater emphasis on time‐related features, with the total time for Task 2 (drawing straight lines) and Task 5 being the most influential. This is illustrated in Figure 4f.

**FIGURE 5:**
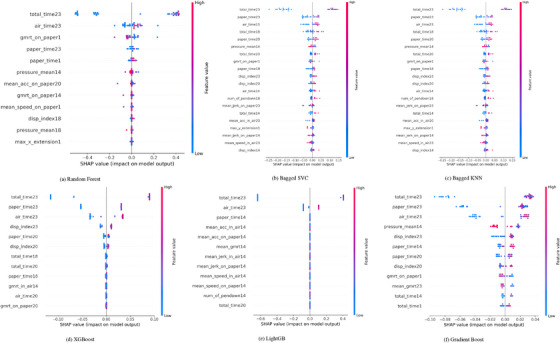
Explanation provided by the SHAP model for memory tasks. (a) Random Forest; (b) Bagged SVC; (c) Bagged KNN; (d) XGBoost; (e) LightGB; (f) Gradient Boost.

The waterfall plots for the memory tasks category, shown in Figure 5, also demonstrate that the RF model continues to prioritize time‐related features, with the total time for task 23 (writing a telephone number under dictation) standing out as the most influential factor. While time‐based features dominate in this category, some additional variables, such as GMRT on paper for task 1 (memorizing and rewriting words), introduce negative SHAP values. This suggests that certain movement characteristics, though less influential than time‐related features, also contribute to the model's predictions (see Figure 5a). Both the Bagged SVC and Bagged KNN models, presented in Figures 5b and 5c, respectively, emphasize the importance of time‐based features, with the total time in Task 23 being particularly impactful for the Bagged SVC model. On the other hand, pressure variance in task 14 (writing under dictation) plays a significant role in Bagged KNN, with the presence of both positive and negative SHAP values. This reflects the nuanced role of fine motor control in memory tasks, where pressure features are significant alongside time‐based attributes.

XGBoost, Gradient Boost and LightGBM, as shown in Figure 5d–f, follow a pattern that aligns with the trends observed in the all‐tasks and copy‐tasks categories. The total time for task 23 remains the dominant feature, with positive SHAP values strongly influencing Alzheimer's classification. Other variables, such as GMRT, pressure mean, and dispersion, display SHAP values close to zero, indicating a minimal impact on the model's predictions in comparison to the time‐related features. This reinforces the overwhelming influence of temporal aspects in the prediction of outcomes within the memory tasks category.

## 4 Discussion

### 4.1 Task‐Specific Performance of Ensemble Methods

The performance of ensemble methods varied across handwriting tasks due to differences in task‐specific requirements and model mechanisms. For example, Bagging methods, such as Bagged KNN and Bagged SVC, demonstrated superior specificity in Copy and Graphic Tasks (92.9% and 89.5%, respectively, Table 3), suggesting their robustness in correctly identifying negative cases. This aligns with Bagging's strength in reducing variance by aggregating predictions from multiple models trained on random subsets of data. In contrast, RF and Gradient Boost methods consistently achieved high sensitivity across Memory and All Tasks. For instance, RF achieved a sensitivity of 88.6% in All Tasks (Table 4), reflecting its ability to handle class imbalance and identify positive cases effectively. Gradient Boost and XGBoost also excelled in tasks with diverse feature spaces, benefiting from their iterative training and adaptive weighting mechanisms. However, Bagged SVC struggled with sensitivity in Memory Tasks (42.0%, Table 3), likely due to its reliance on support vectors, which may not perform well in scenarios with overlapping or less distinct classes. This limitation highlights the importance of selecting ensemble methods based on task‐specific requirements, such as maximizing sensitivity or specificity.

### 4.2 Interpretation of Model Performance

The total time spent on task 23 consistently emerges as the most influential feature across models and task types, particularly for time‐sensitive ensemble models such as RF, XGBoost and Gradient Boost. Results indicate a clear trend: lower total times, specifically within 0 to 10,000, correspond to SHAP values near ‐0.3, indicating a negative contribution to predictions. As task duration exceeds 10,000, SHAP values shift positively, reflecting a corresponding increase in model output. Low total times decrease predictions, especially when paired with low variance in pen‐tip pressure during task 25. Conversely, higher task durations and greater pressure variance show a synergistic relationship, significantly influencing model performance. These findings highlight the critical role of extended durations and pressure variability in enhancing predictive accuracy for AD or similar conditions.

Time‐related features were consistently the most influential predictors across all tasks and models. Task 23 duration, involving writing a telephone number under dictation, was a key indicator of cognitive decline, appearing prominently across all six models. Other features, such as air time (time before task initiation) and paper time (time spent on motor execution), also emerged as relevant predictors, reflecting the dual aspects of cognitive planning and motor execution.

In copying tasks, total time and air time were particularly significant, emphasizing the role of planning in cognitive processes. Increased air time correlated strongly with AD classification, suggesting delays in task initiation and planning due to cognitive difficulties. Paper time, measuring motor execution duration, was less impactful, indicating that cognitive planning—captured by air time—is a more effective predictor of cognitive impairment in these tasks. Similarly, in memory tasks, the total time for task 23 stood out, reinforcing the link between cognitive decline and prolonged task durations. The consistency of time‐based metrics across models underscores their value as reliable markers for assessing AD progression.

Pressure variance and mean pressure emerged as significant indicators of motor control and cognitive function, particularly in copying and writing tasks. For example, pressure variability in tasks such as joining two points with a vertical line (task 3) and writing a sequence of lowercase “i” in cursive (task 8) provided valuable insights into fine motor control. Elevated pressure variability is often correlated with motor difficulties associated with AD. Models like Bagged SVC and Bagged KNN highlighted the critical role of pressure variability in distinguishing individuals with AD from healthy controls. Although pressure variance was less prominent in memory‐based tasks compared to time‐related features, it contributed to overall predictions in tasks like 14 (writing under dictation) and 23. The interplay between pressure and time metrics illustrates the multifaceted nature of AD progression, where both cognitive and motor functions are impaired.

Movement‐related features were less frequent but gained importance in tasks requiring spatial and motor precision, particularly in copying tasks. For example, mean jerk, mean speed and mean speed in‐air were significant predictors in tasks like 14 and 23. Higher jerk values, associated with healthier outcomes, reflect precision in movement. Features such as the dispersion index, measuring spatial extent, were prominent in tasks like 20 and 23, with higher values indicating spatial cognition deficits typical of AD. Similarly, the number of pen‐downs in tasks like 19 (copying a postal order) highlighted participants' difficulty maintaining continuous writing, reflecting cognitive and motor impairments. In graphic tasks, movement‐related features like mean jerk and acceleration on paper were particularly relevant. For instance, in Task 24 (Clock Drawing Test), these features consistently appeared across SHAP graphs, underscoring their importance in evaluating spatial awareness and motor control.

The SHAP analysis consistently showed that time‐related features had the most positive impact on predictions, with longer durations linked to higher AD classification probabilities. Pressure‐related features, with both positive and negative SHAP values, offered insights into motor difficulties. Movement‐related features, particularly in graphic tasks, highlighted the broader effects of AD on spatial awareness and motor control. Together, these findings underscore the importance of integrating time, pressure and movement features for a comprehensive approach to AD prediction.

## 5 Limitations and Future Research

While this study highlights the promising potential of handwriting as a biomarker for early AD detection, offering a non‐invasive and cost‐effective screening methods, several limitations must be considered. First, the limited sample size and diversity of the dataset's cohort may restrict the generalizability of our findings, emphasizing the need for studies with larger and more diverse populations. Additionally, the cross‐sectional nature of the study prevents us from establishing causal relationships between handwriting characteristics and AD progression, underscoring the importance of longitudinal studies. Our focus on specific handwriting tasks may also narrow the scope of our findings, pointing to the need for a more comprehensive evaluation across various cognitive domains.

Moreover, the complexity of our ML models, coupled with potential selection biases and the lack of external validation, raises concerns about generalizability. While SHAP values provide valuable insights, their interpretation can be subjective and context‐dependent, highlighting the need for complementary methods to enhance model interpretability. Additionally, although ensemble methods such as RF, XGBoost and Gradient Boosting have shown effectiveness in this study, the increasing prominence of deep learning techniques like deep neural networks (DNN), CNN and recurrent neural networks (RNN) in similar prediction tasks suggests that future research could benefit from incorporating these more recent models. These techniques have demonstrated superior performance in capturing complex data patterns and could potentially offer improvements in predictive accuracy. Addressing these limitations in future research will be crucial for fully understanding the potential of handwriting as a biomarker for AD detection and monitoring.

## 6 Conclusion

This study examines cognitive assessment through handwriting data, employing interpretable ensemble ML methods to identify key predictors of AD. By analyzing tasks ranging from simple graphic tasks to more complex memory and copy tasks, the study provides significant insights into the relationship between task performance and cognitive decline. The findings reveal that time‐related features are consistently important across all tasks, capturing both the duration of task completion and the time allocated to planning and execution phases. Additionally, pressure‐related features emerge as critical for understanding motor control and cognitive function, highlighting the relevance of motor assessments in AD patient classification. Movement‐related features also serve as vital indicators of spatial cognition and motor control. Variations in spatial accuracy and movement smoothness are significant predictors of cognitive decline, reflecting the spatial and motor impairments associated with AD.

Ensemble methods such as RF, Gradient Boosting and XGBoost demonstrate robust performance in predicting AD based on task‐related features. These methods effectively integrate diverse feature types—including time, pressure and movement into a cohesive predictive model. In the copy tasks, time‐related features and pressure variance were particularly prominent, with increased task duration and higher pressure variance signaling cognitive and motor control difficulties. Interestingly, simpler graphic tasks exhibited high discriminative power, underscoring their sensitivity to early cognitive and motor changes. Features such as Mean Jerk and acceleration during drawing the tasks provided valuable insights into fine motor control and spatial awareness. This study illustrates the efficacy of combining SHAP analysis with ensemble methods to identify key aspects of cognitive decline in AD, as reflected in handwriting data.

## Author Contributions

**Ngoc Truc Ngan Ho**: formal analysis, methodology, visualization. **Paulina Gonzalez**: investigation, methodology, writing–original draft. **Gideon K. Gogovi**: conceptualization, methodology, resources, software, supervision, validation, writing–review and editing.

## Conflicts of Interest

The authors declare no conflicts of interest.

## Data Availability

The datasets used and/or analyzed during the current study are publicly available.
